# N-Acetyl Cysteine as an Adjunct in the Treatment of Tuberculosis

**DOI:** 10.1155/2020/5907839

**Published:** 2020-04-30

**Authors:** Dawit A. Ejigu, Solomon M. Abay

**Affiliations:** ^1^Department of Pharmacology, St Paul's Hospital Millennium Medical College, Addis Ababa, Ethiopia; ^2^Department of Pharmacology and Clinical Pharmacy, College of Health Sciences, Addis Ababa University, Addis Ababa, Ethiopia

## Abstract

Oxidative stress is a common feature of tuberculosis (TB), and persons with reduced antioxidants are at more risk of TB. TB patients with relatively severe oxidative stress had also more advanced disease as measured by the Karnofsky performance index. Since adverse effects from anti-TB drugs are also mediated by free radicals, TB patients are prone to side effects, such as hearing loss. In previous articles, researchers appealed for clinical trials aiming at evaluating N-acetyl cysteine (NAC) in attenuating the dreaded hearing loss during multidrug-resistant TB (MDR-TB) treatment. However, before embarking on such trials, considerations of NAC's overall impact on TB treatment are crucial. Unfortunately, such a comprehensive report on NAC is missing in the literature and this manuscript reviews the broader effect of NAC on TB treatment. This paper discusses NAC's effect on mycobacterial clearance, hearing loss, drug-induced liver injury, and its interaction with anti-TB drugs. Based on the evidence accrued to date, NAC appears to have various beneficial effects on TB treatment. However, despite the favorable interaction between NAC and first-line anti-TB drugs, the interaction between the antioxidant and some of the second-line anti-TB drugs needs further investigations.

## 1. Introductions

Oxidative stress is a common feature of tuberculosis (TB) [1] and is evidenced by elevated lipid peroxidation products such as malondialdehyde (MDA) as well as reduced antioxidant capacity. Compared to healthy individuals, TB patients have low vitamins A, C, and E, selenium, and glutathione (GSH) amounts [2, 3]. TB patients in developing countries have even worse oxidative stress compared with their counterparts in developed nations [2]. After successful treatment with anti-TB drugs, the elevated oxidative stress in TB patients returns to normal [1]. Individuals with diminished antioxidant capacity and increased oxidative stress are also predisposed to TB [4], and TB patients with relatively advanced oxidative stress are more likely to have a severe form of the disease as measured by the Karnofsky performance index [2].

Anti-TB drugs induce several adverse effects in TB patients, and oxidative stress is implicated in mediating these adverse effects. Drug-induced liver injury (DILI) [5] and hearing loss [6] are some of these untoward effects that follow treatment with anti-TB drugs, and both adverse effects are believed to be mediated through oxidative stress. TB patients have already high oxidative stress and hence are prone to these side effects. Hence, antioxidants could potentially mitigate adverse effects induced by anti-TB drugs and facilitate recovery from TB.

Researchers previously appealed for clinical trials aiming at evaluating N-acetyl cysteine (NAC) in attenuating hearing loss in multidrug-resistant TB (MDR-TB) patients owing to the gravity of the problem [7, 8]. However, before testing NAC in TB patients for its hearing loss protective effect, considerations of how NAC would impact other aspects of TB treatment are critical. Unfortunately, there are no such comprehensive scientific reports on NAC and hence this manuscript reviews the broader effects of NAC on TB treatment. This paper discusses NAC's effect on mycobacterial clearance, hearing loss, and DILI as well as the antioxidant's interaction with anti-TB drugs. The safety of NAC itself is not covered here as it has been reviewed elsewhere [7].

## 2. Antimycobacterial Properties of N-Acetyl Cysteine

NAC demonstrated antimycobacterial properties in previous studies of different models [9–11]. NAC cleared mycobacteria through several mechanisms including immunomodulation [12], enhancement of GSH level [13], and direct antimycobacterial effects [14].

### 2.1. Antimycobacterial Mechanisms of NAC

NAC is a precursor of GSH [15], and GSH, in turn, has demonstrated direct antimycobacterial effects. NAC is deacetylated, and the resulting cysteine is used to synthesize GSH [15]. GSH has both direct and indirect antimycobacterial effects. The direct effect of GSH includes enhancing the effect of nitric oxide (NO), one of the immune effector molecules produced by the immune effector cells. Normally, NO had a short-lived antimycobacterial effect but when it is combined with GSH and forms S-nitrosoglutathione (GSSNO) [16], it was persistently released from GSSNO and this prolonged its antimycobacterial effect [17]. Additionally, GSH, being a thiol-containing molecule, might also create a redox imbalance in the mycobacteria since the bacteria use mycothiol [18, 19] as an antioxidant, eventually causing growth inhibition. Another antimycobacterial effect of GSH is through enhancing immune cell activity and cytokine production [20].

NAC also has a direct antimycobacterial effect [14] independent of GSH as well as its free radical-scavenging effects. NAC maintained similar antimycobacterial effects in the presence and absence of the nicotinamide adenine dinucleotide phosphate (NADPH) system in a study that used a knockout mouse model [14]. Had NAC exclusively depended on GSH for its antimycobacterial effect, the absence of NADPH could have diminished its antimycobacterial effect since the recycling of GSH from its oxidized form to its reduced form needs NADPH [21]. In *Escherichia coli*, intracellular cysteine-induced reactive oxygen species (ROS) leading to DNA damage [22] and a similar mechanism of cysteine were confirmed later in mycobacterium [23]. Moreover, cysteine and any other thiol-containing molecules prevented mycobacterial persistence, a state where the bacteria decrease its metabolic rate and become refractory to killing by anti-TB drugs [23]. By avoiding persistence, NAC can also facilitate sterilization by anti-TB drugs and prevent drug resistance.

Moreover, NAC modulated immunity against tuberculosis where it increased interleukin-2 (IL-2), IL-12, and interferon gamma (INF-*γ*) production [9, 20, 24] and these cytokines are important for suppressing mycobacterial proliferation. Additionally, NAC reduced the production of IL-10, a cytokine that favors mycobacterial proliferation [10, 11]. The antioxidant also reduced the proinflammatory cytokines such as IL-1, IL-6, and tumor necrosis factor alpha (TNF-*α*), cytokines known to exacerbate oxidative stress. NAC also increased the immunological activities of various cells including natural killer (NK) cells and macrophages against mycobacterium [13, 25].

### 2.2. Evidence from Studies Other than the In Vitro Model

NAC demonstrated antimycobacterial effects in animal studies [14, 26]. In guinea pigs infected with *Mycobacterium tuberculosis* (MTB), 60 days of oral NAC treatment partially restored red blood cell GSH concentrations and serum total antioxidant capacity. NAC also reduced spleen mycobacterial load as well as lesion burden and the severity in the lungs [26]. However, in this study, NAC did not reduce the mycobacterial load in the lungs. A different study in C57BL/6 mice NAC significantly reduced the mycobacterial load in the lungs after 7 days of treatment with the antioxidant [14].

The difference in the two studies, concerning the reduction in lung mycobacterial load, could be due to species variation, the difference in infection methodology, and the duration of infection. Guinea pigs are very susceptible to infection by MTB and clearing the mycobacteria may be difficult for them while mice are resistant to mycobacteria [27]. The study in guinea pigs also used aerosolization for infecting the animals while the mouse study used intratracheal inoculation. Moreover, the guinea pig study assessed the effect of NAC at 30 and 60 days after infection while the mice study assessed the effect only seven days postinoculation.

NAC also demonstrated several beneficial effects including facilitation of mycobacterial clearance in a clinical trial. In a double-blind, randomized clinical trial of 67 newly diagnosed TB patients, NAC as an adjunct to anti-TB drugs improved smear conversions 3 weeks after initiation of DOTs (directly observed therapies) [28]. NAC at an oral dose of 1200 mg/day resulted in a smear conversion rate of 95.8%, while the conversion rate was 58.3% in the control group. At the end of two months, smear conversion rates were 100% for the NAC group and 91.7% for the control group. Additionally, NAC improved weight gain and response to treatment as assessed by radiology. However, the attrition rate in this study was very high (28%). Two additional trials trying to assess the efficacy and safety of NAC in newly diagnosed TB patients are ongoing (NCT03281226, NCT03702738).

Despite numerous *in vitro* animal studies and a clinical trial which reported antimycobacterial effects of NAC, studies by Khameneh et al. and Vilchèze et al. did not confirm the antimycobacterial effects of the antioxidant in their experiments [23, 29] (Table 1). According to Khameneh et al., NAC did not show antimycobacterial effects against H37Rv even at a concentration of up to 40 mg/ml. However, both studies reported that NAC increased the antimycobacterial effects of anti-TB drugs. What is common in these two studies is that they used TB culture media, Middlebrook 7H9 Broth (7H9) and Lowenstein Jensen (LJ) media, in reporting the sole effect of NAC against TB. From these observations, we can estimate that for its direct anti-TB effect, NAC will probably need macrophages or other similar immune cells, and in the absence of these cells, it can only enhance the effects of other anti-TB drugs.

### 2.3. Interaction with Anti-TB Drugs

NAC showed additive/synergistic antimycobacterial interaction with all first-line and some second-line anti-TB drugs [10, 24, 30]. Administration of NAC and suboptimal concentrations of isoniazid (INH) or rifampicin (RIF) to peripheral blood mononuclear cells (PBMCs) infected with mycobacterium completely cleared mycobacterium from the culture [24]. According to this study, NAC also enhanced the antimycobacterial effect of ethambutol (EMB) in a statistically significant manner. When NAC and anti-TB drugs were also added to the cell culture of macrophages infected with *MTB*, NAC further reduced the viability of the bacteria in the macrophages [10]. In this study, the interaction of NAC with INH, RIF, EMB, and pyrazinamide (PZA) resulted in more than a fourfold further reduction of the CFU. Moreover, coadministration of NAC with clofazimine, bedaquiline, and Q203, an investigational agent, to a culture medium inoculated with *MTB* resulted in the sterilization of the culture medium [30]. According to the study, in the absence of NAC, the three drugs were not able to sterilize the culture media.

The synergistic effect of NAC with INH in clearing mycobacterium could probably be a paradox in light of previous findings on how INH works [31, 32]. INH is a prodrug requiring oxidative activation by the catalase-peroxidase hemoprotein, *KatG*, and the activation of INH by *KatG* is enhanced in the presence of a superoxide. This enhancement is evidenced by an observation that plumbagin and clofazimine, which are superoxide generators, increased the antimycobacterial effect of INH [31]. As an antioxidant, NAC reduces the free radical level, and hence, the antimycobacterial effect of INH could be expected to diminish when coadministered with NAC.

As mentioned earlier, NAC prevents a state of TB persistence, and through this mechanism, the antioxidant has the potential to reduce the duration of anti-TB treatment, reduce the rate of relapse, and prevent resistance against anti-TB drugs [23, 33, 34]. Persisters are subpopulations of the *MTB* colonies which are metabolically inactive and respond poorly to anti-TB drugs. These subpopulations are also the reason for prolonged treatment with anti-TB drugs and an important source of drug resistance. Persisters also do contribute to TB relapses [33]. According to Valchèze et al., the administration of exogenous reducing substances, such as NAC, switched persisters to metabolically active bacteria [23].

Despite synergism of NAC with the first-line anti-TB drugs and clofazimine as well as bedaquiline, assessing NAC's interactions with the remaining second-line drugs is imperative since NAC seemingly antagonized some of the antibiotics [35, 36]. Based on various reports, NAC increased the minimal inhibitory concentrations (MICs) of fluoroquinolones and aminoglycosides against various non-TB bacteria [35]. According to these reports, these effects of NAC are peculiar to the type of organisms since the antioxidant either increased or decreased the MICs depending on the type of organisms [35]. However, Rodriguez et al. explained the counterproductive effects of NAC on the antibiotics' MICs to be due to the acidic pH of the culture medium and adjustment of the medium pH to neutral avoided NAC's negative effect of the antibiotics' MICs [37]. Landini et al. also confirmed that 10 mM of NAC did not adversely affect the MIC of the antibiotics [38]. To further clear the confusion, it would be advisable to test the interaction between NAC and the remaining second-line anti-TB drugs before conducting a clinical trial of NAC in MDR-TB patients.

## 3. Protective Effects of NAC against Ototoxicity

Aminoglycosides are injectable anti-TB drugs used for the treatment of MDR-TB and are associated with ototoxicity [6]. The antibiotics cause loss of hair cells in the cochlea, involved in hearing, and in the vestibular apparatus, involved in maintaining balance. Early damage in the cochlea is limited to the hair cells in the basal region resulting in high-frequency losses in the inaudible range and then involves the apex affecting the low frequencies in the audible range [39]. Initially, the patient's hearing may not be affected but failure to promptly discontinue the antibiotics would diminish the patient's hearing. Ototoxicity caused by aminoglycosides is also irreversible and cumulative [40, 41].

A significant proportion of TB patients undergoing treatment with aminoglycosides develop ototoxicity [6, 42, 43]. Studies suggested that ototoxicity is more frequent in resource-limited settings than in developed nations [39]. The method of diagnosis also has an impact on the proportion of patients identified as having ototoxicity. Diagnosis with high-frequency audiometers is sensitive [44] and gives the true picture of the rates of aminoglycoside-induced ototoxicity than does clinical diagnosis, which underestimates the problem.

Several risk factors are associated with the development of aminoglycoside-induced ototoxicity. Patients with advanced age and low body mass index (BMI) are at a higher risk of ototoxicity [6]. Prolonged duration of treatment with aminoglycosides increases the probability of developing aminoglycoside-induced ototoxicity [44, 45]. Based on a systematic review, MDR-TB patients coinfected with HIV have 22% more risk of their treatment being complicated with ototoxicity [46].

The rate of ototoxicity also varies with the particular aminoglycosides used in the MDR-TB treatment. In a study comparing the rate of ototoxicity induced by capreomycin versus amikacin, the risk of ototoxicity in patients treated with amikacin was increased five times compared with those treated with capreomycin [47]. In another study, in Namibian MDR-TB patients, amikacin was associated with more risk of ototoxicity than kanamycin [48]. Moreover, according to a review on ototoxicity induced by injectable anti-MDR TB drugs, ototoxicity rates caused by streptomycin, kanamycin, and amikacin were 11.8%, 13.3%, and 19.7%, respectively [44]. However, the review seemed to overestimate the ototoxicity rate for capreomycin (25%) probably due to the enrollment in the review of only four patients treated with capreomycin.

NAC attenuated aminoglycoside-induced ototoxicity in various studies [49, 50], and its mechanism of ototoxicity attenuation could be through scavenging free radicals and inhibition of downstream molecular mechanisms of apoptosis induced by oxidative stress. Apart from its free radical-scavenging properties, NAC was shown to inhibit the activation of p38 mitogen-activated protein kinase (MAPK) [51]. The p38 MAPK and its downstream molecular mechanisms, such as activation of caspases and cytochrome c, were also shown to be responsible for aminoglycoside-induced ototoxicity [52].

Aminoglycosides cause ototoxicity through the generation of free radicals and these radicals, in turn, attack the hair cells of the vestibulocochlear nerve [53]. In this process, an iron-aminoglycoside complex is formed and unsaturated fatty acids donate electron in the process of ROS generation [54]. The free radicals cause cochlear and vestibular hair cell loss [55] through apoptosis due to the activation of the p38 MAPK system [56].

In animal studies, enhancing the antioxidant systems and/or free radical-scavenging capacity protected against aminoglycoside-induced ototoxicity. Animals overexpressing superoxide dismutase, the enzyme responsible for scavenging ROS, were resistant to kanamycin-induced ototoxicity [57]. NAC also protected gentamycin- and neomycin-induced ototoxicity in rats and zebrafish [49, 58, 59]. In a rat model which used both NAC and vitamin A for the prevention of gentamycin-induced ototoxicity, both interventions prevented ototoxicity but N-acetyl cysteine showed more protective effect [49]. However, according to an earlier study in guinea pigs by Bock et al., NAC rather worsened kanamycin-induced ototoxicity [60].

NAC protected against aminoglycoside-induced hearing loss in clinical trials of non-TB patients [50, 61, 62] (Table 2). In renal failure patients who developed peritonitis after undergoing peritoneal dialysis and were treated with aminoglycosides, NAC attenuated aminoglycoside induced-ototoxicity. In these studies, gentamycin and amikacin were administered along with NAC for four to six weeks. In a meta-analysis of these clinical trials, NAC protected 86% of the ototoxicity that might have been induced by aminoglycosides [7]. Vural et al. also confirmed that NAC protected against aminoglycoside-induced ototoxicity during a one-month treatment period with the antioxidant [63]. However, the protective effect diminished after one year. In this trial, treatments with aminoglycosides and NAC were completed at the same time. This finding probably suggests that for maximal protection with the antioxidant, we need to continue the administration of NAC for a little longer after completion of aminoglycoside administration. According to pharmacokinetic studies, aminoglycosides accumulate in the hair cells slowly and their half-life in these cells is prolonged [64, 65]. This means aminoglycosides could continue to cause hair cell damages even after they are discontinued.

## 4. Protective Effect of NAC against DILI

DILI is one of the major adverse effects of treatment with anti-TB drugs. Patients treated with anti-TB drugs may experience DILI ranging from simple hepatic enzyme elevations to severe clinical hepatitis. In the presence of hepatitis symptoms, such as abdominal pain, nausea, vomiting, unexplained fatigue, or jaundice, hepatotoxicity is defined as an elevation of liver enzymes, alanine (ALT) and/or aspartate (AST) transaminase, more than three times the upper limit of the normal range (ULN). But in the absence of symptoms, either of the enzymes must be elevated more than five times ULN [66, 67]. The culprits causing DILI among the first-line anti-TB drugs are INH, PZA, and RIF while from the second line drugs, the list includes fluoroquinolones, ethionamide, prothionamide, amoxicillin-clavulanate, and para-aminosalicylic acid [68]. The rate of DILI in TB patients could sometimes go up as high as 27% [69].

DILI could have serious implications on the outcome of TB patient treatment [5, 70]. Some patients with DILI may develop acute liver failure, ascites, and hepatic encephalopathy, and yet others may manifest only with simple enzyme elevations. A study in China demonstrated more than a 9-fold risk of unsuccessful treatment and more than a 2-fold increased risk of prolonged intensive phase treatment in patients with DILI compared to TB patients without DILI [70]. Anti-TB associated DILI could also result in death, and this was more common in patients who developed encephalopathy, ascites, and jaundice [5]. The mortality rate in patients who developed anti-TB-associated DILI could also be as high as 23%.

Different risk factors are associated with the development of anti-TB DILI. Various studies suggested advanced age as one of the risk factors [71, 72] even if other studies did not confirm age as a risk factor [73, 74]. Some studies also indicated HIV to be a risk factor for anti-TB-associated DILI [75, 76]. Additionally, other risk factors associated with anti-TB DILI include low BMI [77, 78], viral hepatitis [79], INH acetylation status [80], and genetic makeup of the patients [81].

NAC attenuates anti-TB DILI through scavenging free radicals formed during the metabolism of the drugs as well as through enhancing the synthesis of GSH [82]. Anti-TB drugs induce liver injury via the formation of free radicals and then the free radicals damage different cellular parts [83]. The free radicals are probably generated during the metabolism of the various anti-TB drugs [67]. Normally, these molecules are detoxified by the host's antioxidant system including GSH. However, if the antioxidant system is compromised for various reasons, the free radicals could damage different structures within the cells and perpetuate the oxidative stress through lipid peroxidation.

**Figure 1:**
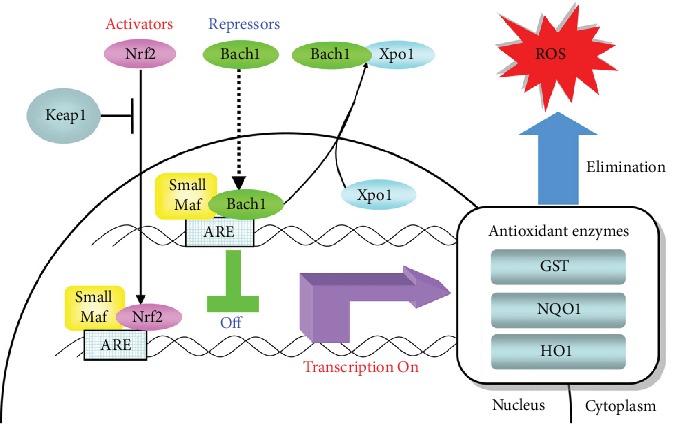
Activator and repressor arms in the antioxidant pathway. Schematic representation indicates the location and translocation of relevant genes involved in the activator arm (Nrf2/small Mafs/Xpo1) and repressor arm (Bach1/small Mafs/Keap1) in the antioxidant pathway as well as the transcriptional regulation of antioxidant enzymes (NQO1/HO1) against oxidative stress in hepatocytes. Nrf2: nuclear factor erythroid 2-related factor 2; Keap1: Kelch-like ECH-associated protein 1; Bach1: BTB and CNC homology 1; Xpo1: exportin 1; ARE: antioxidant-responsive element; GST: glutathione S-transferase; NQO1: NAD (P) H dehydrogenase quinone 1; HO1: heme oxygenase 1; ROS: reactive oxygen species [81].

According to studies on genetic polymorphisms of oxidant-antioxidant systems, mutations leading to reduced antioxidant enzymes or increased prooxidant enzymes resulted in increased susceptibility to anti-TB DILI [81, 84, 85]. A loss of function mutation on *MAFK* encoding MafK (small Maf basic leucine zipper proteins) increased susceptibility to anti-TB DILI [81] (Figure 1). Normally, the binding of MafK with Nrf2 (nuclear factor erythroid 2-related factor 2) leads to the upregulation of antioxidant enzymes. Similarly, a gain of function mutations on *NOS2A*, encoding inducible nitric oxide synthase (iNOS) and *BACH1*, encoding BTB and CNC homology 1 (bach1), increased susceptibility to anti-TB DILI [81]. *NOS2A* encodes the enzyme nitric oxide synthase while *BACH1* encodes Bach1, whose binding with Nrf2 leads to the downregulation of antioxidant enzymes. Additionally, loss of function mutations on *HMOX1*, the gene encoding for heme oxygenase 1 recognized as phase II antioxidant enzyme, and *NQO1*, which encodes NAD (P)H: quinone oxidoreductase 1, were also associated with susceptibility to anti-TB DILI [84]. A meta-analysis also confirmed the association of polymorphisms of GSTM1 and GSTT1, glutathione S-transferase encoding genes, with anti-TB DILI [85].

NAC prevented anti-TB drug-induced hepatotoxicity in animal studies [82, 83] (Table 3). A study in rats demonstrated that INH and RIF each at a dose of 50 mg/kg depleted GSH as well as other antioxidants and increased lipid peroxidation in the liver tissue [83]. The changes in antioxidants levels were also accompanied by histopathologic changes such as portal triaditis, lobular inflammation, and patchy necrosis. NAC at a dose of 100 mg/kg prevented all these pathologic changes and attenuated the ALT elevation from 102 IU/l to 28 IU/l and the AST elevation from 578 IU/l to 165 IU/l at 3 weeks. Another study in rats confirmed the protective effect of NAC on RIF-induced hepatotoxicity. However, the latter study did not evidence a reduction in the level of GSH after treatment with RIF alone [82]. An *in vitro* study in human liver cancer cell line (hepg2) cells also demonstrated a protective property of NAC against anti-TB-induced hepato-/cytotoxicity [86] (Table 3).

A clinical trial of NAC in newly diagnosed TB patients also showed protective effects against hepatotoxicity [87] (Table 3). Sixty patients, aged greater than 60, were recruited in this trial and followed for two weeks. The study was not blinded and controls did not receive placebo. NAC was administered orally at dose of 600 mg twice daily, and the incidence of hepatotoxicity in the control group was 37.5% and none in the NAC group. As the authors themselves acknowledged, these results need to be interpreted with caution and there is a need for well-controlled larger trials with longer follow-up.

## 5. Conclusions

Based on the evidence accrued to date, NAC appears to have various beneficial effects on TB treatment and its evaluation in clinical trials is justifiable. For maximal protection by NAC against anti-TB-induced hearing loss, we need to continue the administration of NAC for a little longer after completion of aminoglycoside administration. Moreover, the interaction between NAC and some of the second-line anti-TB drugs needs further investigations despite favorable interaction between NAC and first-line anti-TB drugs, bedaquiline and clofazimine.

## Figures and Tables

**Table 1:** Summary of studies on anti-TB effects of NAC against *MTB*.

Author	Year	Type of study	Anti-TB effect	Effect measurement	Type of immune cells	Type of *MTB*	Conc. of NAC	Study subjects	Remark	Reference
Venketaraman et al.	2006	Cell culture	Yes	CFU reduction, effect size not indicated	Human Øs	H37Rv	10 mM	HIV +ve and -ve		[9]
Cao et al.	2018	Cell culture	Yes	50% CFU reduction	THP-1	Erdman strain	10 mM	NA	Also showed that anti-TB drugs and NAC have synergistic effects	[10]
Venketaraman et al.	2008	Cell culture	Yes	CFU reduction, effect size not indicated	Human Øs	H37Rv	10 mM	HIV -ve	Used whole blood culture	[11]
Guerra et al.	2011	Cell culture	Yes	CFU reduction, effect size not indicated	Human NK cells	H37Rv	20 mM	HIV +ve and -ve	NK cells pretreated with NAC	[20]
Morris et al.	2013	Cell culture	Yes	CFU against control, effect size not indicated	Human Øs	H37Rv	10 mM	HIV +ve and -ve		[13]
Amaral et al.	2016	Cell culture	Yes	CFU vs. control, effect size not indicated	THP-1	H37Rv, avium, Beijing 1471, M. bovis	10 mM	HIV -ve		[14]
Guerra et al.	2011	Cell culture	Yes	CFU reduction, effect size not indicated	Human Øs	H37Rv	5, 10, 20 mM	HIV +ve and -ve		[20]
Vilchèze et al.	2017	Cell culture & 7H9	No	4-5 log CFU reduction	J774 Øs	H37Rv	5-10 mM	NA	Anti-TB effect when only combined with RIF/INH	[23]
Lamprecht et al.	2016	7H9 medium	Yes	CFU reduction, sterilized	NA	H37Rv	5 mM	NA	Combined with BDQ, Q203, and CFZ	[30]
Teskey et al.	2018	Cell culture & animal study	Yes	CFU reduction	Human PBMCs	Erdman strain	10 mM	Healthy and T2DM patients, rats		[24]
Palanisamy et al.	2011	Animal study	Yes	CFU reduction	NA	H37Rv	400 mg/kg/day for 60 days	Guinea pigs		[26]
Mahakalkar et al.	2017	Clinical trial	Yes	Sputum conversion	NA	NA	600 mg/day	Smear +ve TB patients		[28]
Khameneh et al.	2016	LJ media	No	NA	NA	H37Rv	0.04-40 mg/ml	NA	Anti-TB effect only when combined with RIF or INH	[29]

CFU: colony-forming unit; Øs: macrophages; NK cells: natural killer cells; LJ: Lowenstein Jensen; PBMC: peripheral blood mononuclear cells; T2DM: type 2 diabetes mellitus; NAC: N-acetyl cysteine; INH: isoniazid; RIF: rifampicin; BDQ: bedaquiline; CFZ: clofazimine; Q203: investigational product.

**Table 2:** Clinical trials conducted to investigate the otoprotective effect of 600 mg twice a day NAC against AGs in non-TB patients.

Author	Year	Effect measurement	Duration of protection	Study subjects	Sample size	Type of aminoglycoside	Country of study	Remark	Reference
Feldman et al.	2007	25% vs. 60% in favor of NAC	6 weeks	Hemodialysis patients	40	Gentamycin	Israel		[61]
Tokgoz et al.	2011	3.3% vs. 70% in favor of NAC	4 weeks	Peritoneal dialysis patients	60	Amikacin	Turkey	Amikacin combined with vancomycin	[62]
Kocyigit et al.	2015	In favor of NAC	4 weeks	Peritoneal dialysis patients	46	Amikacin	Turkey		[50]
Vural et al.	2018	In favor of NAC	4 weeks	Peritoneal dialysis patients	40	Amikacin	Turkey	Reassessment after 12 months showed reduced effects	[63]

NAC: N-acetyl cysteine; AGs: aminoglycosides.

**Table 3:** List of studies on the hepatoprotective effect NAC on anti-TB drugs.

Author	Year	Type of study	Types of animals or cells	Dose or concentration of NAC	Type of anti-TB	Protection by NAC	Reference
Attri et al.	2000	Animal study	Wistar rats	100 mg/kg	INH (50 mg/kg), RIF (50 mg/kg)	Yes	[83]
Rana et al.	2006	Animal study	Wistar rats	100 mg/kg	RIF (50 mg/kg)	Yes	[82]
Singh et al.	2012	*In vitro*	HepG2	10 *μ*M	INH (100, 200 mM), RIF (50 mM), PZA (100, 200 mM)	Yes	[86]
Baniasadi et al.	2011	Clinical trial	NA	600 mg PO BID	INH, RIF, PZA, ETH	Yes	[87]

NAC: N-acetyl cysteine; INH: isoniazid; RIF: rifampicin; PZA: pyrazinamide; hepG2: human liver cancer cell line.
